# Information Propagation Formalized Representation of Micro-blog Network Based on Petri Nets

**DOI:** 10.1038/s41598-019-57237-6

**Published:** 2020-01-20

**Authors:** Xun Liang, Shusen Zhang, Yu Liu, Yuefeng Ma

**Affiliations:** School of Information Science, Qufu Normal University, Shandong, 276826 China

**Keywords:** Computer science, Information technology

## Abstract

The description of user behavior in social networks is an important issue for studying social networks. Given that Petri nets can describe the resource flow problem, this study utilizes the features of Petri nets to portray the user behavior states during the message propagation of a micro-blog network and presents an information propagation formalized representation method of a micro-blog network. On this basis, this study analyzed the proposed formalized representation method in detail. We provide examples of applying formalized representation (e.g., micro-blog network addiction of users, user behavior influence, and public opinion analysis). In addition, we introduce the algorithms of formalized representation. We conduct experiments using Sina micro-blog data. Results show that the information propagation formalized representation method of micro-blog network based on Petri nets can depict user behaviors of micro-blog network intuitively and accurately. This study reveals a new perspective for information transmission of a micro-blog network and provides some tools to support public opinion monitoring and micro-blog marketing applications.

## Introduction

With the development of Internet technology and the popularization of network self-media, social networks have become increasingly important in people’s lives, especially the extensively used social network platforms, such as Facebook, Twitter, micro-blog network, and Flickr. These platforms have emerged from different platforms based on the characteristics of diverse groups of people^1^.

Research of a social network can start from the structure, content, and time. The first aspect is to start with the structure of a social network, including the granularity of “point”, “line”, and “surface.” From the “degree” of nodes, this study investigates the specific characteristics of a “point”, that is, the granularity of a “point”. The connections between the nodes in the social network and the paths formed between the nodes can be evaluated. The latter forms the “lines” in the social network. Moreover, the relationship between the lines, that is, the granularity of the “lines”, can be ascertained. In addition, the multiple user groups formed in the social network, which established different communities during information dissemination in the social network and the “face” granularity of the social network, can be assessed. The second aspect is based on social network contents, which includes the attributes, texts, images, audios, and videos of users in the social network. The dissemination and flow of various types of content in social networks comprise the third aspect of social network research, which is a time-based study that analyzes the time of user behavior and the relationship between the users. These abovementioned three aspects constitute the main part of social network research.

Currently, people can receive and publish information anywhere, anytime, which makes information spread faster and wider^2^. The information dissemination problem in social networks slightly reflects the resource flow problem. For example, disseminating micro-blog information in a micro-blog network indicates that a piece of information is transmitted from one user to multiple users and from multiple users to many users in a network structure composed of users. During this period, the disseminated information may be transmitted back to the original user who first published the micro-blog posts through user forwarding, commenting, and other behaviors or to the user who forwarded the micro-blog content, thereby forming the complex “information resource” flow problem in the micro-blog network.

Petri nets^3^ are extensively used in industry research. The dining philosophers problem^4^ is a typical example of resource use in which five philosophers gather around a round table full of food and require chopsticks to eat, with one chopstick between each philosopher. A philosopher must pick up the chopsticks on the two sides simultaneously when he wants to eat the food on the table. The philosopher’s state changes from “thinking” to “eating” when he picks up the chopsticks on the left and right sides. The philosophers only sit and think when the chopsticks are insufficient to eat food. That is, individual users can achieve different state transitions by waiting and occupying resources. The change in philosopher’s behavior state can be described by Petri nets and can simulate the process of philosophers eating concurrently. In addition, a method of system behavior simulation based on color Petri net can be used to provide resources for enterprise software system^5^. In order to solve the traffic congestion problem, a novel regulatory traffic light control system based on synchronized timed Petri nets is proposed, which solves a traffic congestion problem with a regulatory traffic light control technique that is effective in preventing vehicles from entering traffic congestion zones^6^.

To represent the process and situation of information transmission in social networks, people have established various information transmission models of social networks based on the characteristics of information flow over time in the network structure and investigated the information transmission of online social networks from the perspective of models. We use Petri nets to describe and model the social network because they are a tool used for describing distributed systems, which can describe the system structure and simulate the dynamic operation of the system to represent the flow of resources in the structure. Thus, we can intuitively describe the social network during information dissemination in terms of “point”, “line”, and “surface” in the structure, analyze the behavior of users, investigate the content of information, and depict the dynamic process of information dissemination.

Given the abovementioned ideas, this study presents a unified formal representation method of information dissemination in a micro-blog network based on Petri nets. This method is based on the structural characteristics of a micro-blog network and investigates the unified formal representation of Petri nets in a social network from the perspectives of content and time. The proposed method describes and analyzes users’ characteristics during information dissemination from the perspective of user behavior (including posting or forwarding, commenting, replying, liking, deleting, and not taking any action) and the change in a user’s behavior state and describes the dynamic operation of an information dissemination system. On the basis of the unified formal representation method of Petri nets, the characteristics of users in a social network are analyzed, and the analysis of users’ addiction to micro-blog, users’ influence, and network public opinion based on users’ online behavior state is completed.

In social networks, user nodes are an important part of information dissemination. Information can be disseminated in the social network structure given the online behavior of users in the social network. At the same time, information transmission models motivated by the classical epidemic propagation, have been applied to a wide-range of social systems, and the external influence has significant impact on information spreading along with social activities^7^. Existing research of information dissemination models includes information cascade model^8^ based on influence probability, linear threshold model^9^, and virus dissemination model^10^. Among these models, the independent cascade model^11,12^ based on influence probability and the linear threshold model^8,11^ are the two most basic propagation models^13^. The nodes in these models have two states, namely, active and inactive states, and can realize the transition between the two states. In the independent cascade model, each user makes decisions on his own behavior based on the observed behavior of other users^14^. The independent cascade model is required to specify an influence probability *p* for each edge <*u*, *v* > ∈ *E* in directed graph *G*(*V*, *E*) formed by the network structure, thus indicating the probability that node *u* can activate node *v* into an active node through edge <*u*, *v*>. During propagation, node *v* can be activated by active neighbor nodes and become active nodes. Gruhl *et al*.^15^ modified the model to be applicable to actual information transmission with time delay in social networks by considering the probability of each side’s behavior. The linear threshold model, which indicates that the degree of vulnerability of node *v* may be affected, is expressed by the threshold *θ ∈*[0, 1] of each node *v*. A small *θ* denotes a high vulnerable node *v* that will be affected by active neighbor nodes during propagation. At time *q* of propagation, node *v* becomes active at time *q* + 1 when the influence of node *v* is greater than or equal to *θ* by the sum of the influence of the active neighbor node set. Saito *et al*.^16^ proposed an extended model based on the independent cascade and linear threshold models and considered the effect of asynchronous time delay.

In addition, research on information dissemination model includes virus dissemination and the corresponding diffusion models^10,17^. Infectious disease model is mainly based on the representation and distribution of disease in the population. Susceptible–infected (SI) and SI–Recovered (SIR) models are the typical infectious disease model^17^. For the SIR infectious disease model, the basic states include susceptible, infected, and recovered. Individuals in each state change from susceptible to infectious with probability *β* and from infectious to recovery with probability *γ*. Xiong *et al*.^18^ improved the SIR model based on the characteristics of a micro-blog network. A user’s status changes into susceptible, contact, infection, and anti-infection states. A new transmission model was proposed. Muroya *et al*.^19^ and Prakash *et al*.^20^ increased the time factors to improve the epidemic model. Zhan X *et al*. proposed a nonlinear model to further interpret the coupling effect based on the SIS (Susceptible-Infected-Susceptible) model, and a multi-outbreak phenomenon emerges via the effect of coupling dynamics^20^. In addition, the study of information dissemination model includes the dynamic study of information dissemination based on game theory knowledge^21^ and the simulation model of public opinion dissemination based on game theory^22^.

Most existing studies contain dynamic characteristics of information dissemination of social networks. For example, Bian *et al*.^23^ predicted the spread of micro-blog information based on the proposed diffusion target influence model. Iribarre *et al*. focused on the impact of human behavior on dynamic information dissemination patterns^24^. In addition, the main contents of online social network information dissemination research include the effect of user behavior on influence^25^, popularity prediction of user forwarding behavior on information^26^, analysis of user behavior in social networks, and identification of user identity through structural and temporal characteristics.

Petri nets, as a tool for describing the running process of a system, have been investigated in social network and information dissemination mechanism. The present study uses a micro-blog network as an example to investigate the unified formal representation of user behavior description based on Petri nets during information dissemination. In comparison with the abovementioned studies, our study presents a unified formal representation of information dissemination based on user behavior and combines user behavior with Petri net theory. Moreover, this study describes user behavior during information dissemination of micro-blog network combined with the changes in user status and provides a formal representation of information dissemination in a micro-blog network based on Petri nets.

## Formal Representation of Petri Nets in Micro-blog Networks

### Micro-blog network characteristics and related definitions

A micro-blog network is a social network with numerous users. Each user can take different actions in the network (including posting/forwarding, liking, commenting, replying, and deleting). The main contents of this study are the information dissemination and user behavior characteristics in a micro-blog network. The information dissemination in a micro-blog network is investigated from the perspective of user behavior state. Any user can participate in the interaction and dissemination of micro-blog information on the network, and users can generate various behavior states through interactive behavior during information dissemination. The basic concepts used in the unified formal representation are defined as follows.

**Definition 1** (Micro-blogging Repository *s*). In a micro-blog network, each user *u*’s operation behavior state *a*_*i*_ (including no action *a*_0_, posting or forwarding *a*_1_, liking *a*_2_, commenting *a*_3_, replying *a*_4_, and deleting *a*_5_; assume that *i* = 0, 1, 2, 3, 4, 5, respectively) is called a micro-blog repository (in a circular form and referred to as repository), that is, a repository *s*_*k,i*_ represents the current behavior state *a*_*i*_ of user *u*_*k*_ (where *k* = 1, 2, 3, …).

**Definition 2** (Micro-blog Transition *t*). Micro-blog transitions (referred to as transitions) indicate that users focus on the specific operation behavior of behavior state changes, that is, *t*_*k*,*i*_ → _*w*,*j*_ (*k*, *w* represent the users; *i*, *j* represent the values of different behavior states of users; transitions are represented by a square). A kind of transition represents the behavior that causes the change in operation behavior state among different users, thereby forming the main line *L*_1_. For example, the information is transmitted from User 1 to User 2. User 2 receives the information and forwards the micro-blog. The expression method of transition is *t*_1,1_ → _2,1_. Another kind of transition represents the behavior that causes the changes between the user’s own operation behavior states, thus forming the main line *L*_2_. At this time, the transition between forwarding and commenting statuses is expressed as *t*_2,1_ → _2,3_ when User 2 comments on the information that he/she forwarded.

**Definition 3** (Micro-blog Token). A flag in a repository (a black spot in the repository) indicates that the user has taken a certain action. The number of tokens in a repository indicates the number of times a user takes certain actions in a given time. During information dissemination, the behavior of users at different times may be repeated, that is, a user may take the same behavior at different times. Therefore, for each user’s behavior state *a*_*i*_, vector *a*_*i*_ (*q*_1_, *q*_2_, *q*_3_, …, *q*_*m*_) denotes that the time for users to adopt *a*_*i*_ is *q*_1_, *q*_2_, *q*_3_, …, *q*_*n*_. The number of time *q*_*m*_ is recorded as *n*_*ki*_, which indicates the number of actions *a*_*i*_ of user *k*.

**Definition 4** (Weight Function *W*). The input weight function *W*(*s*, *t*) of transition *t* is 0, that is, it consumes 0 token and output weight function *W*(*s*, *t*) = 1, that is, one token is generated every time a user takes an action.

**Figure 1:**
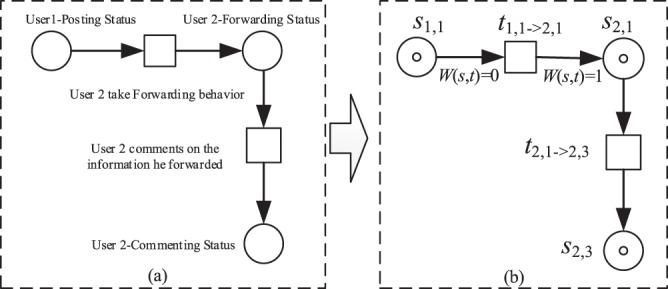
Example of the transition relationship between micro-blog network information dissemination repositories.

Taking the information dissemination illustrated in Fig. 1(a) as an example, User 1 publishes the micro-blog, User 2 sees the information and exhibits the forwarding behavior, and User 2 comments on the micro-blog information he/she has forwarded. Figure 1(b) plots the basic relationships among micro-blog repositories, micro-blog transitions, micro-blog logos, and weight functions based on Fig. 1(a). Among these relationships, *s*_1,1_ denotes that User 1 has posted micro-blog posts, and *t*_1,1_ → _2,1_ indicatesthat User 2 sees the information and exhibits the forwarding action. Moreover, *s*_2,1_ indicates that User 2 has forwarded the micro-blog posts in its current state, and *t*_2,1_ → _2,3_ represents that User 2 has commented on the information that he/she forwarded.

**Definition 5** (Conditions for Micro-blog Changes). Transition *t* occurs when users in a micro-blog network take the following behavior.

**Definition 6** (User Behavior Vector). In a micro-blog network, let each user *u* form a 6 dimensional vector, which is *a*_*k*_ = (*a*_0_, *a*_1_, *a*_2_, *a*_3_, *a*_4_, *a*_5_)^T^ with six basic behaviors (no action *a*_0_, posting or forwarding *a*_1_, liking *a*_2_, commenting *a*_3_, replying *a*_4_, and deleting *a*_5_) in a micro-blog. When the user’s behavior has temporal characteristics, the user’s behavior vector is expressed as *a*_*k*_ = (*a*_0_(*n*_*k*0_), *a*_1_(*n*_*k*1_), *a*_2_(*n*_*k*2_), *a*_3_(*n*_*k*3_), *a*_4_(*n*_*k*4_), *a*_5_(*n*_*k*5_))^T^, where T denotes transposition.

**Definition 7** (User Behavior Matrix, UBehavior). A matrix consisting of user behavior vectors is called a user behavior matrix, which is denoted as UBehavior = [*a*_0_(*n*_*k*0_), *a*_1_(*n*_*k*1_), *a*_2_(*n*_*k*2_), *a*_3_(*n*_*k*3_), *a*_4_(*n*_*k*4_), *a*_5_(*n*_*k*5_)] _6×*N*_, where each column is a user behavior vector. For *N* users in the micro-blog network, each user may have six behavior actions, which forms *N* columns of the user behavior matrix and then a 6 × *N* order micro-blog network user behavior matrix.

**Definition 8** (Identification Status M). In a micro-blog social network, information is disseminated from time to time, where users in a micro-blog may take the following behavior at any time. After users take actions, the network state changes accordingly. Corresponding to the social network Petri nets, identity state *M* represents the number of tokens per repository in the current network, that is, the number of different behaviors taken by each user is counted.

## Unified Formal Representation of Information Dissemination in Petri Nets

In a micro-blog network, information is transmitted among users in the network. With the passage of time, information will spread to different users, users will take different actions to participate in information dissemination, and each behavior state of users will affect the means of information dissemination. On the basis of the abovementioned definition, in a social network, a state of each user’s following behavior forms a repository in Petri nets, and the connection between repositories in the formal representation of Petri nets is based on the user structure in the actual network. Based on the behavior state and the change between user’s behavior states, we provide a formal Petri net representation of micro-blog network information dissemination from the perspective of Petri nets.

**Figure 2:**
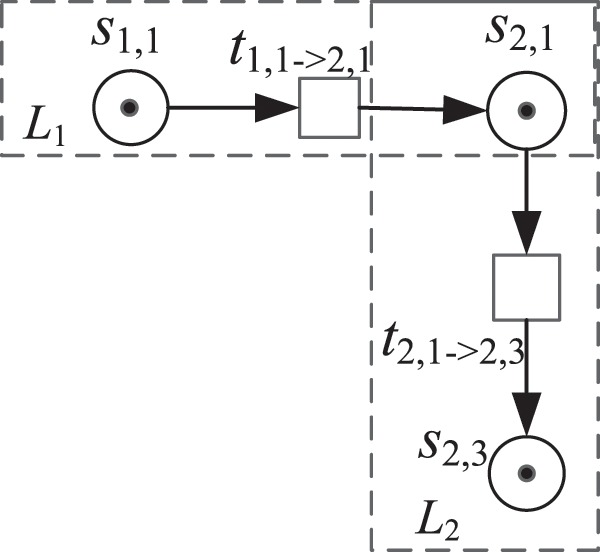
Brief description of the formal representation of Type I Petri nets.

**Definition 9** (Type I Petri Nets Formalized Representation). Σ = (S, T, F, W, K, M) is the basic Petri net formalized representation of micro-blog networks. Among these variables, (*S*, *T*, *F*) is a standard Petri net triple, *W*: *F* →  {0, 1} indicates the weight function, *K*: *S* → ∞ represents the capacity function of the repository, and *M*: *S* → ∞ denotes an identifying state of Σ. Evidently, Σ only contains the basic relationship between the state of a user’s action and the change in state. Thus, we consider this type of formal representation of Petri nets as Type I Petri net representation. Therefore, the formal representation of Type I Petri nets can be simply described as follows: User 1 publishes or forwards micro-blog posts, User 2 forwards User 1’s micro-blog information, and the relationship between users’ behavior and state changes. Mainline *L*_1_; User 2 comments on his forwarded micro-blog information, thus forming the main line *L*_2_ of the user’s own behavior state change relationship. Figure 2 depicts a formal representation of forwarding and commenting between Users 1 and 2. The user behavior state and the transition relationship between states in a micro-blog network are composed of several main lines of *L*_1_ and *L*_2_. Therefore, Type I Petri nets can be composed of six behavior states of each user in the network and the transition relationship between them and form a formal representation of Petri nets in Type I micro-blog network.

The above initial representations of information dissemination and user behavior in a micro-blog network described by the basic pattern of Petri nets only represent the basic elements of a micro-blog network. They ignore many important features of information dissemination, such as the time dynamics during information dissemination. In addition, with the development of Internet technology, mobile terminals have become inevitably popular. Geographic location information is gradually and extensively used on social websites. Therefore, the formal representation of Type I Petri nets must be expanded and optimized to describe the information dissemination and user behavior of a micro-blog network completely and accurately. For the sake of narrative convenience, we first define the temporal characteristics.

**Definition 10** (Petri Timestamp of Micro-blog Network). The time point in information dissemination is called Petri time stamp (*q*) of a micro-blog network formalized by Petri nets. The adding repository set of Petri timestamp in a micro-blog network (referred to as timestamp) can be expressed as the time point when user *k* takes action *i*, marked as ${s}_{{\rm{k}},{\rm{i}},{q}_{{\rm{ki}}}}$, and the adding transition set can be expressed as ${t}_{{\rm{k}},{\rm{i}},{q}_{{\rm{ki}}}\to {\rm{w}},{\rm{j}}{q}_{{\rm{wj}}}}$ (*k*, *w* indicate the user, and *i*, *j* denote the user’s behavior state).

**Definition 11** (Type II Petri Nets Formalized Representation). Σ = (*S*, *T*, *F*, *W*, *K*, *M*, *Q*) is the formal representation of Type II Petri nets in social networks. Among these variables, (*S*, *T*, *F*, *W*, *K*, *M*) is the formal representation of Type I Petri nets, *Q* is the time function defined on the set of repositories, and the set of transitions, *Q*: *S*  →  *R*, *T* → *R*_0_ × *R*_0_, where *R*_0_ represents the set of non-negative real numbers.

This representation includes the state of a user’s action and behavior and the relationship between them and increases the time characteristics of social network information dissemination simultaneously. Therefore, we call the formal representation of Type I Petri nets with time stamps Type II Petri nets.

**Figure 3:**
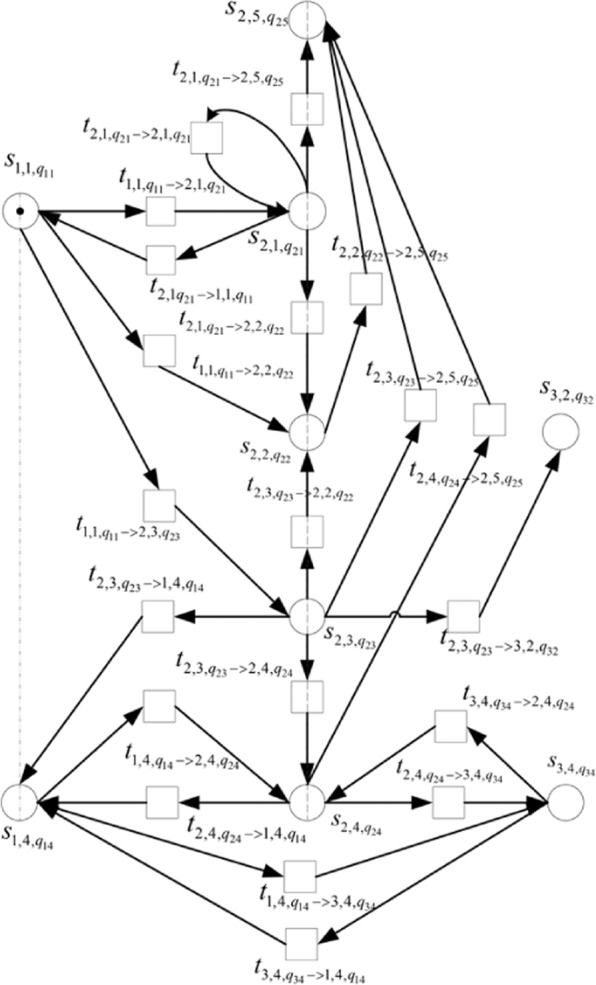
Formal representation and description of Type II Petri nets.

Given the above definition, the formal representation of Type II Petri nets in micro-blog information transmission is described, as demonstrated in Fig. 3.

In Fig. 3, Type II Petri nets formally represent various possible user behaviors during information dissemination. ${s}_{1,1,{q}_{11}}$ is formed when User 1 posts at *q*_11_. At *q*_21_, User 2 can forward User 1’s micro-blog information posted at time *q*._11_, that is, through transition ${t}_{1,1,{q}_{11}\to 2,1{q}_{21}}$. Similarly, User 2 can like, reply, comment, or delete the information forwarded by himself at other time points and interact with User 1 and 3. Based on the formal representation of Type II Petri nets, the description of the repository and the meaning of the transition in the micro-blog information transmission exhibited in Fig. 3 are summarized in Tables 1 and 2.

**Table 1:** Explanation of Micro-blog Repository Meaning Formalized by Type II Petri Nets.

Micro-blog repository	Meaning of micro-blog repository
${s}_{1,1,{q}_{11}}$	User 1’s forwarding or posting behavior state at time *q*_11_
${s}_{1,4,{q}_{14}}$	User 1’s response behavior state at time *q*_14_
${s}_{2,1,{q}_{21}}$	User 2’s forwarding or posting behavior state at time *q*_21_
${s}_{2,2,{q}_{22}}$	User 2’s liking behavior state at time *q*_22_
${s}_{2,3,{q}_{23}}$	User 2’s commenting behavior state at time *q*_23_
${s}_{2,4,{q}_{24}}$	User 2’s response behavior state at time *q*_24_
${s}_{2,5,{q}_{25}}$	User 2’s deleting behavior state at time *q*_25_
${s}_{3,2,{q}_{32}}$	User 3’s liking behavior state at time *q*_32_
${s}_{3,4,{q}_{34}}$	User 3’s response behavior state at time *q*_34_

**Table 2:** Explanation of Micro-blog Transition Meaning Formalized by Type II Petri Nets.

Micro-blog transition	Meaning of micro-blog transition
${t}_{1,1,{q}_{11}\to 2,1,{q}_{21}}$	At *q*_21_, User 2 forwards User 1’s micro-blog information
${t}_{1,1,{q}_{11}\to 2,2,{q}_{22}}$	At *q*_22_, User 2 likes the micro-blog information posted or forwarded by User 1 at *q*_11_
${t}_{1,1,{q}_{11}\to 2,3,{q}_{23}}$	At *q*_23_, User 2 comments on the micro-blog information posted or forwarded by User 1 at *q*_11_
${t}_{1,4,{q}_{14}\to 2,4,{q}_{24}}$	At *q*_24_, User 2 replies to User 1’s response at *q*_14_
${t}_{1,4,{q}_{14}\to 3,4,{q}_{34}}$	At *q*_34_, User 3 replies to User 1’s response at *q*_14_
${t}_{2,1{q}_{21}\to 1,1,{q}_{11}}$	At *q*_11_, User 1 forwards User 2’s micro-blog information posted at *q*_21_
${t}_{2,1,{q}_{21}\to 2,1,{q}_{21}}$	At *q*_21_, User 2 forwards his own micro-blog message
${t}_{2,1,{q}_{21}\to 2,2,{q}_{22}}$	At *q*_22_, User 2 likes the micro-blog information that he posted or forwarded.
${t}_{2,1,{q}_{21}\to 2,5,{q}_{25}}$	At *q*_25_, User 2 deletes his own micro-blog information posted or forwarded at *q*_21_
${t}_{2,2,{q}_{22}\to 2,5,{q}_{25}}$	At *q*_25_, User 2 deletes the micro-blog information that he posted or forwarded
${t}_{2,3,{q}_{23}\to 2,2,{q}_{22}}$	At *q*_22_, User 2 likes the micro-blog information that he commented at *q*_23_
${t}_{2,3,{q}_{23}\to 2,4,{q}_{24}}$	At *q*_24_, User 2 replies to his own reviews at *q*_23_
${t}_{2,3,{q}_{23}\to 1,4,{q}_{14}}$	At *q*_14_, User 1 replies to User 2’s reviews at *q*_23_
${t}_{2,3,{q}_{23}\to 2,5,{q}_{25}}$	At *q*_25_, User 2 deletes his own reviews at *q*_23_
${t}_{2,3,{q}_{23}\to 3,2,{q}_{32}}$	At *q*_32_, User 3 likes User 2’s reviews at *q*_23_
${t}_{2,4,{q}_{24}\to 1,4,{q}_{14}}$	At *q*_14_, User 1 replies to User 2’s reviews at *q*_24_
${t}_{2,4,{q}_{24}\to 3,4,{q}_{34}}$	At *q*_34_, User 3 replies to User 2’s response at *q*_24_
${t}_{2,4,{q}_{24}\to 2,5,{q}_{25}}$	At *q*_25_, User 2 deletes his own reply at *q*_24_
${t}_{3,4,{q}_{34}\to 1,4,{q}_{14}}$	At *q*_14_, User 1 replies to User 3’s response at *q*_34_
${t}_{3,4,{q}_{34}\to 2,4,{q}_{24}}$	At *q*_24_, User 2 replies to User 3’s response at *q*_34_

The formal representation of Type II Petri nets in a social network with time stamps can dynamically describe the user’s operation behavior and change characteristics in information dissemination. However, many other factors, such as geographical location, text content, photo information, video and audio, exist in an actual social network. These factors form the richness and complexity of a social network. On this basis, we provide Definitions 12–14 as follows.

**Definition 12** (Type III Petri Nets Formalized Representation). Σ = (*S*, *T*, *F*, *W*, *K*, *M*, *Q*, *G*) is the formal representation of Type III Petri nets. Among these variables, (*S*, *T*, *F*, *W*, *K*, *M*, *Q*) is the formal representation of Type II Petri nets, *G* is the geographic location information defined on the collection of databases that represent the geographic location information where users are operating, and *R*_1_ is the longitude information in the geographic location, with a range of [−180, 180],which indicate the eastern and western hemispheres by positive and negative signs, correspondingly.*R*_2_ is the latitude information of the geographic location, with a range of [−90, 90], which indicate the southern and northern hemispheres by positive and negative signs, respectively, *G*: *S* → *R*_1_ × *R*_2_.

Similarly, feature values, such as the emotional value of text content and the number of words posted to the formal representation, must be added when investigating the text information in social networks. Therefore, we provide Definition 13 as follows.

**Definition 13** (Type IV Petri Nets Formalized Representation). Σ = (*S*, *T*, *F*, *W*, *K*, *M*, *Q*, *G*, *V*) is the formal representation of Type IV Petri nets. The formal representation of type IV Petri nets is a formal representation of Type III Petri nets using the text sentiment value *V*.

We can define additional formal representations of Petri nets of dimensions through analogy.

**Definition 14** (Type *z* Petri Nets Formalized Representation). The dimension of formal representation of Type *z* Petri nets is based on the formal representation of Type IV Petri nets of Σ = (*S*, *T*, *F*, *W*, *K*, *M*, *Q*, *G*, *V*) by adding *z*−3 dimensions to form the formal representation of Type *z* Petri nets of a micro-blog network.

Given limited space, the following part of this paper only discusses the formal representation of Type II Petri nets.

## Typical Application Scenario Examples of a Formal Representation of Type II Petri Nets

The unified formal representation of information dissemination in a micro-blog network can describe the user’s behavior flexibly from the structure, content, and time of the micro-blog. We provide an example of the formal description of the actual micro-blog information dissemination based on the actual situation in the micro-blog network.

Sina Weibo user “Xiaoli Colleagues Said” forwarded post p on August 29, 2016, and user “turbosun” liked the post on August 30, 2016, forwarded the micro-blog post at another time on the same day, and commented on the micro-blog he forwarded at the third time point of the day.

On the basis of the route and time of transmission, we can extract the information into the formal representation of Type II Petri nets, and the relevant user and user behavior states are displayed in Table 3.

**Table 3:** Related Meaning of Micro-blog Instances Based on Type II Petri Net Formalized Representation.

Name	Meaning
User 1	Weibo User “Xiaoli Colleagues Said”
User 2	Weibo User “turbosun”
Micro-blog repository ${s}_{1,1,{q}_{11}}$	“Xiaoli Colleagues Said” posted on August 29, 2016
Micro-blog repository ${s}_{2,1,{q}_{21}}$	On August 30, 2016, “turbosun” forwarded “Xiaoli Colleagues Said’s” post on August 29, 2016.
Micro-blog repository ${s}_{2,2,{q}_{22}}$	On August 30, 2016, “turbosun” liked “Xiaoli Colleague Said’s” post.
Micro-blog repository ${s}_{2,3,{q}_{23}}$	On August 30, 2016, “turbosun” commented on the post that he forwarded.
Micro-blog transition ${t}_{1,1,{q}_{11}\to 2,1,{q}_{21}}$	On August 30, 2016, “turbosun” forwarded “Xiaoli Colleagues Said’s” post on August 29, 2016.
Micro-blog transition ${t}_{1,1,{q}_{11}\to 2,2,{q}_{22}}$	On August 30, 2016, “turbosun” liked “Xiaoli Colleague Said’s” post on August 29, 2016.
Micro-blog transition ${t}_{2,1,{q}_{21}\to 2,3,{q}_{23}}$	“turbosun” commented on its forwarded post on August 30, 2016

**Figure 4:**
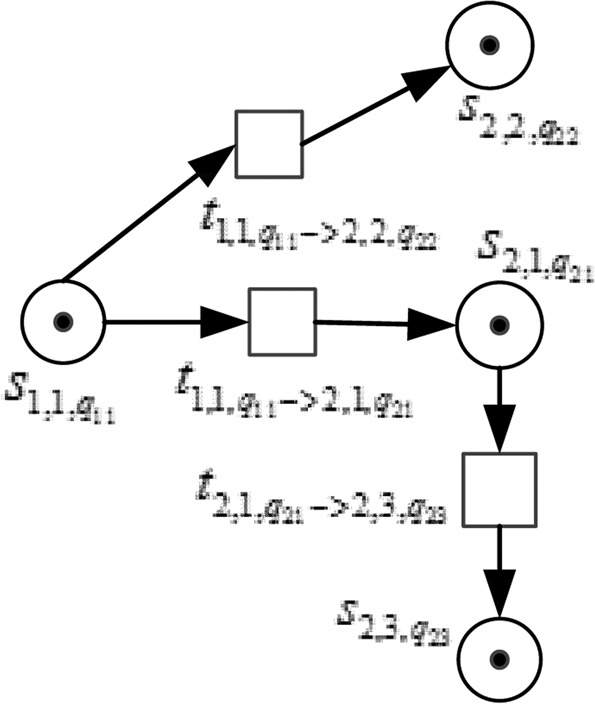
Micro-blog network instances based on Type II Petri net formalized representation.

The relationship between users’ status is complicated when we use words to describe the content of information dissemination in a micro-blog network. Thus, the graphical representation is formalized by Type II Petri nets after “Xiaoli Colleagues Said” forwarded post *p*. The behavior status and interaction between netizens “turbosun” and “Xiaoli Colleagues Said” on August 30, 2016, are exhibited in Fig. 4.

The information dissemination between “Xiaoli Colleagues Said” and “turbosun” described by the formal representation of Type II Petri nets shows the situation between the different states of the two users and their relationship in the structure. For content, the dissemination of information at different times can be described intuitively.

Next, we discuss the application of micro-blog network formalized representation by taking users’ addiction to a micro-blog network, public opinion situation of the micro-blog network, and the influence of users’ behavior as examples.

### Micro-blog network addition

With the development and extensive use of the Internet, an increasing number of people are involved in the network, and the number of people using social networks has increased. Most research on people’s addiction to Internet behavior are based on medicine or psychology. The behavior is called Internet addiction or tendency of Internet addiction. Considering that Internet addicts cannot control their online behavior, Internet addiction has aroused widespread concern in society, thus resulting in many health or social problems^27^.

The degree of addiction of users in social networks generally depends on two aspects. On the one hand, this condition comes from the influence of users’ followers or fans on users, including browsing and participating in the content published by other users in the social network. On the other hand, this condition comes from users’ own participation behavior, such as posting in the social network. Generally, these conditions constitute user behavior in a social network. Therefore, the user’s behavior in the social network can be evaluated to determine whether a user has a certain degree of addiction to the micro-blog network. Based on the formal representation of Type II Petri nets, we define micro-blog network addiction, as indicated in Definition 15. Moreover, users’ addiction to micro-blogging can be determined on the basis of other types of Petri nets. For example, we can determine micro-blog addiction from the perspective of text information based on the formal representation of Type IV Petri nets (such as the number of words posted).

**Definition 15** (Micro-blog Network Addition). In a given time (*t*_0_, *t*_*D*_) (*t*_0_ < *t*_*D*_), if the *t*otal number of actions of user *k* in time (*t*_0_, *t*_*D*_) is grea*t*er than *η*, then user *k* has micro-blog network addiction, where *η* is a given constant in advance. If the number of actions of a user is expressed as *n*_*ki*_ (*i* = 0, 1, 2, 3, 4, 5), then it is expressed as ${\sum }_{i=0}^{5}{n}_{ki} > \eta$.

Thus, if the frequency of user *k*’s behavior in a given unit time is greater than *η*/(*t*_*D*_ − *t*_0_), then the user has a micro-blog network addiction.

If “Xiaoli Colleagues Said” demonstrates the following behavior in a micro-blog network for a long time, and the total number of user behavior reaches a certain value in a period, then we consider that “Xiaoli Colleagues Said” demonstrates micro-blog network addiction.

### Analysis of sum public opinion

User participation in a micro-blog network affects the development direction and trend of micro-blog public opinion information. User behavior can slightly reflect the current situation of the development of an event. We can analyze the public opinion situation of a social network from identification state *M* formally expressed by Petri nets based on user behavior.

**Definition 16** (Public Opinion Situation). In micro-blog network information within a given time (*t*_0_, *t*_*D*_) (*t*_0_ < *t*_*D*_), the to*t*al behavior state of all participants, called the sum public opinion in the micro-blog network information, is calculated. The calculation method is expressed as follows:

$${\rm{Sum\; Public\; Opinion}}=\mathop{\sum }\limits_{k=1}^{x}\mathop{\sum }\limits_{{\rm{i}}=0}^{5}{n}_{{\rm{ki}}},$$

where *n*_*ki*_ indicates the number of tokens of microblog library for behavior state (*a*_*i*_) of user *k*, and *x* denotes the number of users’ participating in the event.

For example, if we analyze the public opinion situation of the micro-blog information forwarded by “Xiaoli Colleagues Said”, then we can calculate the sum of users’ behavior states. Based on the micro-blog case illustrated in Fig. 4, the public opinion situation was 4 at 15:50 on August 30, 2016.

### User behavior influence

The influence of micro-blog users has attracted considerable attention in the field of micro-blog research. User influence includes the influence of user’s micro-blog content, the degree of user’s influence on followers, the influence of user’s micro-blog radiation range, and the influence of user’s active and passive behaviors^28^. The present study measures the influence of user’s active behavior through user’s online active behavior (including posting or forwarding, commenting, liking, replying, and deleting) and provides the definition and calculation method of user’s active behavior influence from the perspective of Type II Petri nets.

**Definition 17** (User Active Behavior Influence). In a given time (*t*_0_, *t*_*D*_) (*t*_0_ < *t*_*D*_), the to*t*al ratio of the number of *a*_*i*_ (publish_*i*_ng or forwarding, commenting, liking, replying, and deleting) for each user *u*’s behavior to the time interval of the behavior state itself is called behavior influence. The calculation method based on Petri nets is expressed as follows:

$${\rm{Behavior\; Influence}}=\mathop{\sum }\limits_{i=0}^{5}\frac{{n}_{{u}_{i}}}{{q}_{i,{\rm{last}}}-{q}_{i,{\rm{first}}}},$$

where ${n}_{{u}_{i}}$ denotes the number of occurrences of user *u*’s type *i* behavior, and *q*_*i*,last_ and *q*_*i*,first_ denote the last and the earliest occurrence times of user *u*’s type *i* behavior. The number of occurrences is set to 1 when a certain behavior only occurs once.

In the example of micro-blog information dissemination demonstrated in Fig. 4, the influence of user “turbosun’s” active behavior is 3.

## Unified Formal Representation Algorithms for Type II Petri Nets

For the information dissemination of a micro-blog network, network structure, user behavior characteristics, and information content are the key factors that constitute the dynamic information dissemination. In this study, we focus on user behavior, micro-blog information, and micro-blog network structure and comprehensively consider all the aspects of the proposed formal representation of information dissemination in a Type II micro-blog network. Simultaneously, the application algorithm of the formal representation of Type II Petri nets is presented on the basis of two micro-blog information transmission units, namely, event and topic.

### User behavior-centered algorithms

In the micro-blog network information dissemination, information is transmitted through a user’s forwarding behavior. We can analyze users’ basic behavior from different angles based on the formal representation of micro-blog information dissemination by Petri nets. By formalizing the Petri nets representation of users’ behavior, we can analyze the characteristics of individual user’s communication behavior in micro-blog information dissemination, discover user’s behavior preferences, and analyze user’s participation in information dissemination. Furthermore, we can analyze the influence of users in the network, find the users with micro-blog addiction in the micro-blog network, and discover interesting contents for users.

For the six main behaviors of users in information dissemination, we can use the following algorithms to analyze the quantitative preference characteristics of user behavior.

**Algorithm 1:**
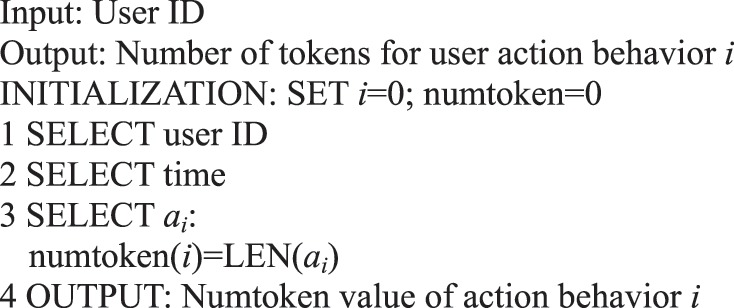
(Quantity of Single User Action Behavior *i*).

**Algorithm 2:**
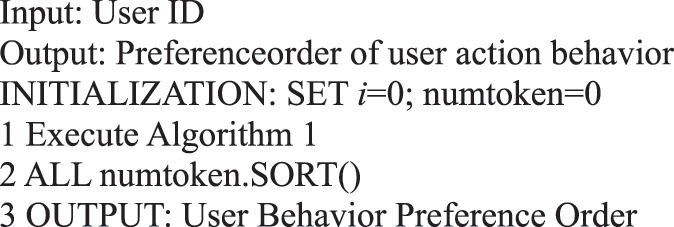
(Preference Algorithm of Single User Action Behavior).

**Algorithm 3:**
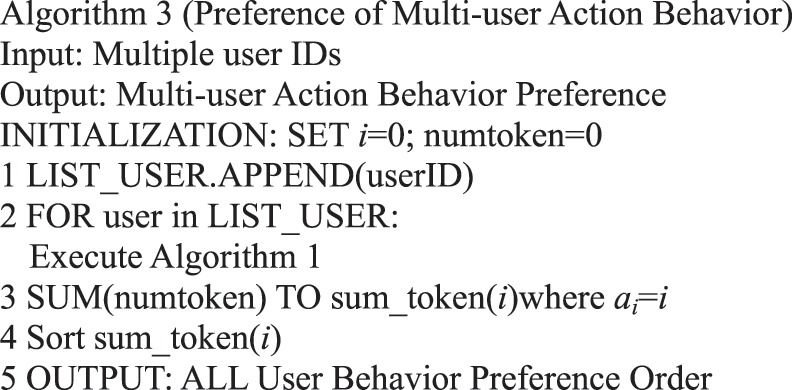
(Preference of Multi-user Action Behavior).

We can analyze the user’s response to the event in micro-blog information dissemination, the performance of a single user’s followers in micro-blog information dissemination, and the followers’ behavior preferences and provide Algorithm 4.

**Algorithm 4:**
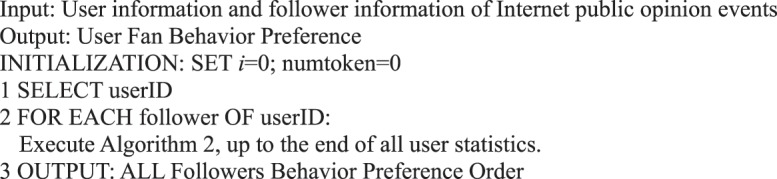
(Preference of User’s Follower Action Behavior).

In addition to analyzing users’ basic behavior in information dissemination, we provide an algorithm that corresponds to the formal representation of Petri nets from the perspective of the specific application illustrated previously. For example, we must determine the number of tokens in the repository of the user’s behavior in a certain period to determine whether a user is addicted to a micro-blog network in a certain behavior, as presented in Algorithm 5.

**Algorithm 5:**
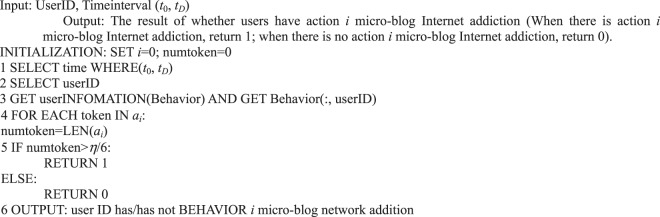
(Micro-blog Internet Addiction of Single User Action *i*).

**Algorithm 6:**
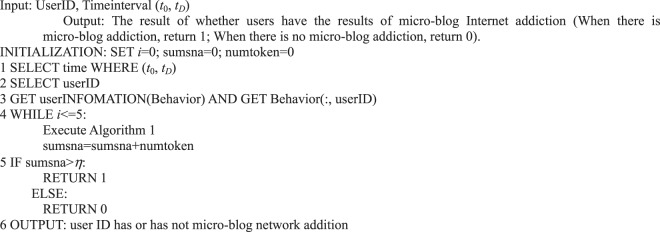
(Micro-blog Internet Addiction of Single User).

In the information dissemination of a micro-blog network, the participation degree of users is the main content of our user behavior analysis, that is, the activity degree of users. From the behavioral perspective, user activity is related to the number of posts, comments, replies, and likes. This study measures user activity from the perspective of user behavior, regardless of the weight of each action on the activity. We use the sum of the number of posts/forwards, comments, replies, and likes as the user activity.

**Algorithm 7:**
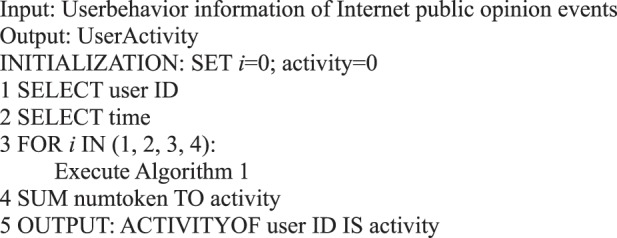
(Single User Activity).

During information dissemination, different users play diverse roles in the direction and situation of information dissemination, that is, the influence of user’s behavior is different. Based on the definition of a user’s active behavior influence provided in Definition 17, we present a correlation analysis algorithm of a user’s active behavior influence based on Petri nets.

**Algorithm 8:**
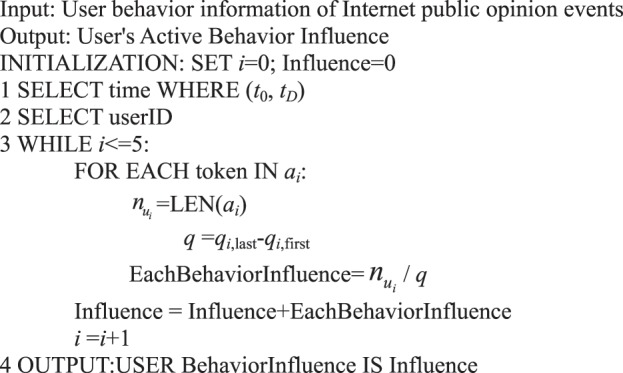
(Single User Active Behavior Influence).

In a micro-blog network, user similarity is related to a user’s attribute information^29^. In this study, we analyze the similarity between user interaction behaviors. The behavior similarity between adjacent users is calculated on the basis of the user’s posting/forwarding and commenting behaviors. The calculation method is expressed as follows^29–32^:

$$\begin{array}{l}{\rm{Interaction}}={w}_{1}\ast [{\rm{retweet}}\_{\rm{num}}(u,v)+{\rm{retweet}}\_{\rm{num}}(v,u)]/2\\ \,+{w}_{2}\ast [{\rm{comment}}\_{\rm{num}}(u,v)+{\rm{comment}}\_{\rm{num}}(v,u)]/2\\ \,+{w}_{3}\ast [{\rm{reply}}\_{\rm{num}}(u,v)+{\rm{reply}}\_{\rm{num}}(v,u)]/2\end{array}$$

The influence of weight is disregarded in this study, and *w*_1_ = *w*_2_ = *w*_3_ = 1/3.

**Algorithm 9:**
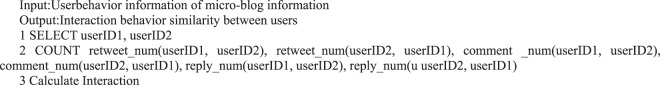
(User Interaction Behavior with Adjacent Transmitters).

Through the above-mentioned algorithm for analyzing the basic behavior of users, we can obtain certain help and reference in analyzing user influence, discovering Weibo Internet addiction users and calculating user similarity.

### Algorithms centered on micro-blog information

In contrast to the formal representation centered on the user behavior in a micro-blog, the research on information dissemination centered on the content of micro-blogs that focus on the overall dissemination trend of information, such as the scope and cycle of information dissemination. The formal Petri net representation of information content can express the public opinion situation of micro-blog information dissemination. We obtain Algorithm 10 in accordance with Definition 16.

**Algorithm 10:**
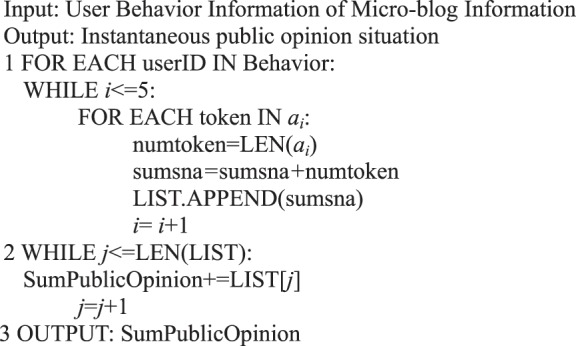
(Instantaneous Public Opinion Situation of Single Micro-blog Information).

**Algorithm 11:**
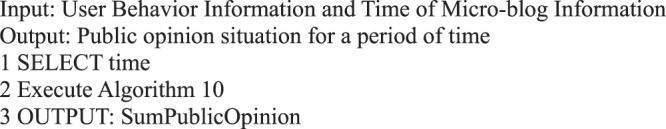
(Public Opinion Situation for a Period of Time).

The information dissemination algorithm centered on Weibo information content has important value for the analysis of the scope of information dissemination and the public opinion of information dissemination.

### Centering on network structure

In addition, we use the proposed formal representation of Type II Petri nets from the perspective of micro-blog network structure to describe micro-blog information dissemination path analysis and micro-blog information dissemination formed by community analysis.

For the path analysis, we analyze the critical path, the path statistics, the shortest path in micro-blog information dissemination, and the path with the least nodes.

**Algorithm 12:**
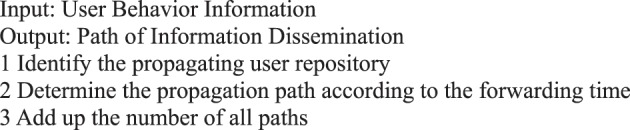
(Propagation Path of Single Micro-blog Information).

**Algorithm 13:**
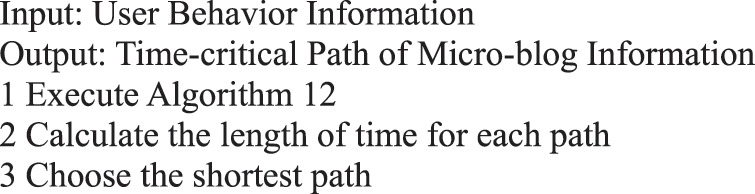
(Time-Critical Path of Single Micro-blog Information).

**Algorithm 14:**
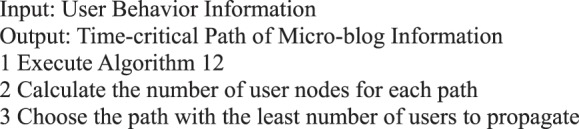
(Critical Path of Single Micro-blog Information Node).

**Algorithm 15:**
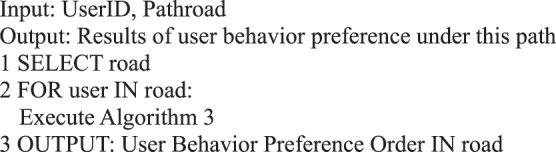
(Action Behavior Preference of Single Path Users).

The algorithm based on the perspective of microblog network structure has certain help and practical value for analyzing the microblog information propagation path and analyzing the microblog community.

### Algorithms that synthesize different angles

Information disseminated in social networks is transmitted to multiple users. We can determine users’ micro-blog addiction because a message travels in different paths.

**Algorithm 16:**
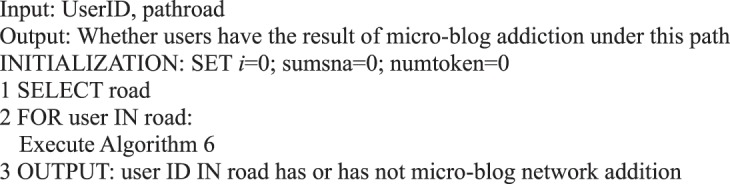
(Micro-blog Internet Addiction of Single Path Users).

**Algorithm 17:**
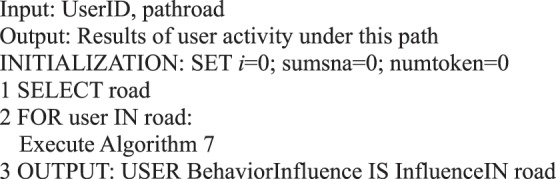
(Activation of Single Path Users).

**Algorithm 18:**
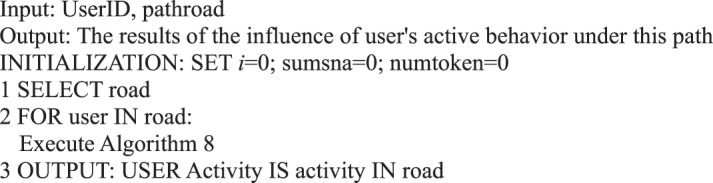
(Active Behavior Influence of Single Path User).

**Algorithm 19:**
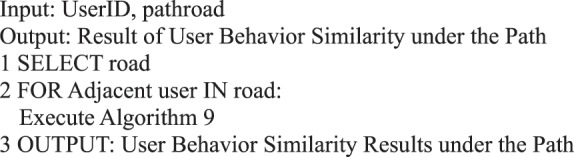
(Behavior Similarity of Single Path Adjacent User).

**Algorithm 20:**
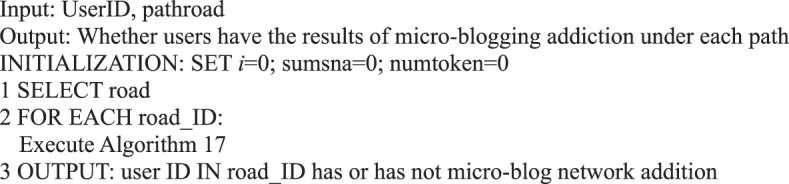
(Micro-blog Internet Addiction of Multipath Users).

**Algorithm 21:**
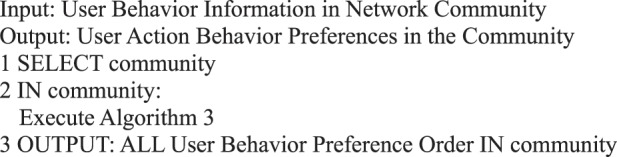
(User Action Preference in Community).

**Algorithm 22:**
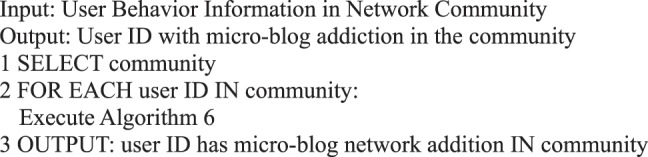
(User Micro-blog Internet Addiction Algorithm in Community).

**Algorithm 23:**
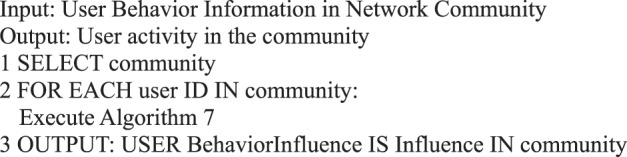
(User Activity in Community).

**Algorithm 24:**
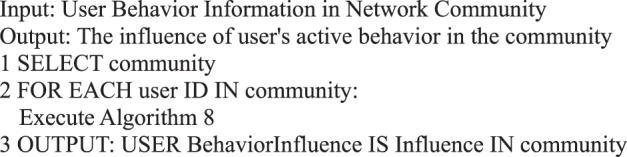
(Influence of User’s Active Behavior in the Community).

**Algorithm 25:**
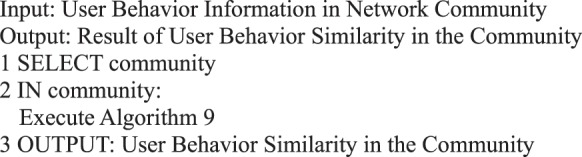
(User Behavior Similarity in Community).

Similarly, we can judge the public opinion situation in a community when the social network is divided into different communities.

**Algorithm 26:**
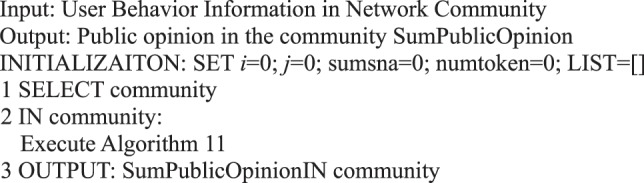
(Public Opinion Situation in Community).

### Algorithms based on different micro-blog information transfer units

We must count the behavior of all users in an event when we analyze their behavior preferences. We can determine the online behavior that users tend to perform within a specified time when an event or message is sent. Based on the judgment of the situation of network public opinion, for different events or topics in social networks, users may have diverse preferences in participating in an event or topic. Therefore, we can model and analyze each event by Petri net formal representation to examine the behavior status of users in various events. We can determine the user’s preference for a period by identifying a user’s response to an event.

**Algorithm 27:**
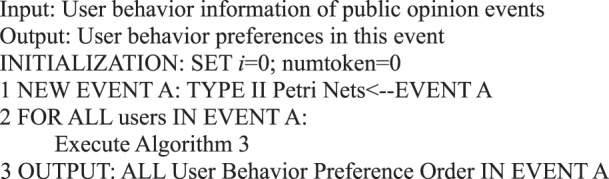
(Behavior Preference of Single Event Users).

Therefore, a user behavior matrix can be constructed for Events A and B by analyzing the behavior of the same user in the two events. A calculation can be performed on the basis of the following matrix after determining the user activity under the two events.

In social network public opinion events, each event forms a user behavior matrix. For Events A and B, the behavior of the same user in the two events is analyzed. Let behavior matrix A_UBehavior and B_UBehavior be expressed as follows:

$$\begin{array}{c}{\rm{A}}\_{\rm{UBehavior}}=[\begin{array}{cccc}{a}_{0}({n}_{10}) & {a}_{0}({n}_{20}) & {a}_{0}({n}_{30}) & \ldots \\ {a}_{1}({n}_{11}) & {a}_{1}({n}_{21}) & {a}_{1}({n}_{31}) & \ldots \\ {a}_{2}({n}_{12}) & {a}_{2}({n}_{22}) & {a}_{2}({n}_{32}) & \ldots \\ {a}_{3}({n}_{13}) & {a}_{3}({n}_{23}) & {a}_{3}({n}_{33}) & \ldots \\ {a}_{4}({n}_{14}) & {a}_{4}({n}_{24}) & {a}_{4}({n}_{34}) & \ldots \\ {a}_{5}({n}_{15}) & {a}_{5}({n}_{25}) & {a}_{5}({n}_{35}) & \ldots \end{array}],\\ {\rm{B}}\_{\rm{UBehavior}}=[\begin{array}{cccc}{a}_{0}({m}_{10}) & {a}_{0}({m}_{20}) & {a}_{0}({m}_{30}) & \ldots \\ {a}_{1}({m}_{11}) & {a}_{1}({m}_{21}) & {a}_{1}({m}_{31}) & \ldots \\ {a}_{2}({m}_{12}) & {a}_{2}({m}_{22}) & {a}_{2}({m}_{32}) & \ldots \\ {a}_{3}({m}_{13}) & {a}_{3}({m}_{23}) & {a}_{3}({m}_{33}) & \ldots \\ {a}_{4}({m}_{14}) & {a}_{4}({m}_{24}) & {a}_{4}({m}_{34}) & \ldots \\ {a}_{5}({m}_{15}) & {a}_{5}({m}_{25}) & {a}_{5}({m}_{35}) & \ldots \end{array}].\end{array}$$

When user behavior matrix A_UBehavior and user behavior matrix B_UBehavior are user behavior matrices with user participation in two events, a new user behavior matrix C_UBehavior  =  A_UBehavior + B_UBehavior can be obtained to indicate the user activity of these users in the two events. This matrix is expressed as

$${\rm{C}}\_{\rm{UBehavior}}=[\begin{array}{cccc}{a}_{0}({m}_{10}+{n}_{10}) & {a}_{0}({m}_{20}+{n}_{20}) & {a}_{0}({m}_{30}+{n}_{30}) & \ldots \\ {a}_{1}({m}_{11}+{n}_{11}) & {a}_{1}({m}_{21}+{n}_{21}) & {a}_{1}({m}_{31}+{n}_{31}) & \ldots \\ {a}_{2}({m}_{12}+{n}_{12}) & {a}_{2}({m}_{22}+{n}_{22}) & {a}_{2}({m}_{32}+{n}_{32}) & \ldots \\ {a}_{3}({m}_{13}+{n}_{13}) & {a}_{3}({m}_{23}+{n}_{23}) & {a}_{3}({m}_{33}+{n}_{33}) & \ldots \\ {a}_{4}({m}_{14}+{n}_{14}) & {a}_{4}({m}_{24}+{n}_{24}) & {a}_{4}({m}_{34}+{n}_{34}) & \ldots \\ {a}_{5}({m}_{15}+{n}_{15}) & {a}_{5}({m}_{25}+{n}_{25}) & {a}_{5}({m}_{35}+{n}_{35}) & \ldots \end{array}],$$

where *m*_*ki*_ + *n*_*ki*_ denotes the number of behavioral *a*_*i*_ of user *k*.

We summarize the abovementioned process as Algorithm 28.

**Algorithm 28:**
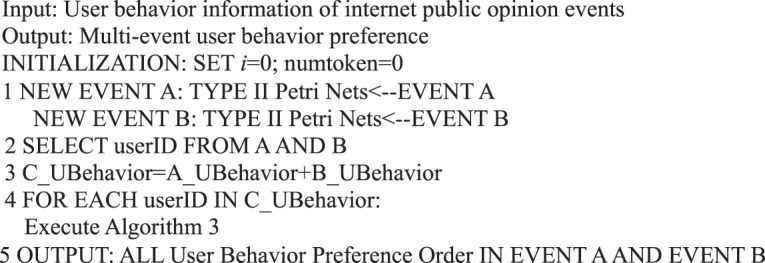
(Multi-Event User Behavior Preference).

**Algorithm 29:**
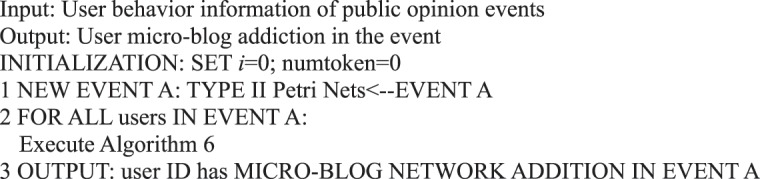
(Micro-blog Internet Addiction of Single Event Users).

**Algorithm 30:**
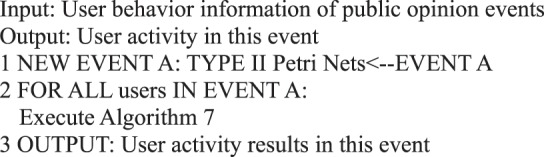
(Single Event User Activity).

**Algorithm 31:**
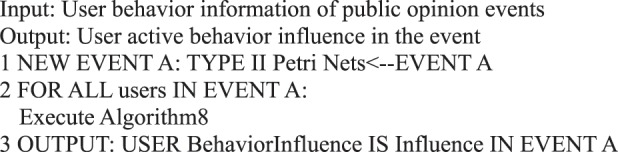
(Active Behavior Influence for Single Event Users).

**Algorithm 32:**
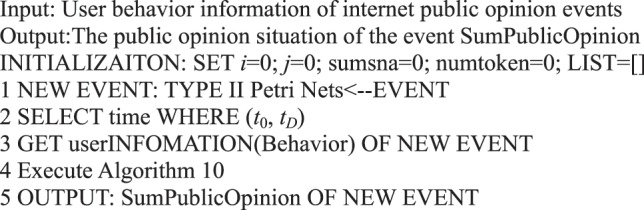
(Single Event Public Opinion Situation).

**Algorithm 33:**
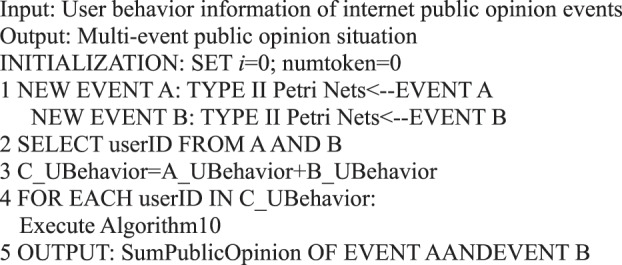
(Multi-event Public Opinion Situation).

**Algorithm 34:**
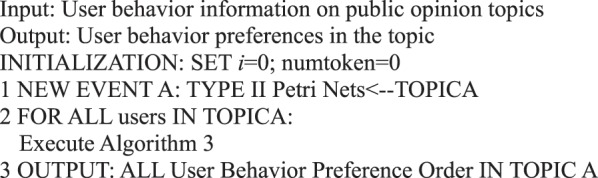
(Single Topic User Action Behavior Preference).

**Algorithm 35:**
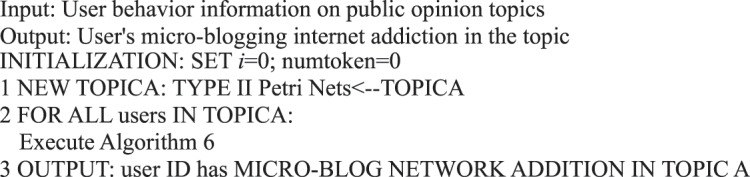
(Micro-blog Internet Addiction of Single Topic Users).

**Algorithm 36:**
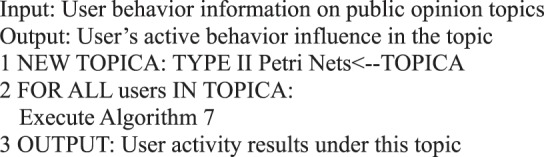
(Single Topic User Activity).

**Algorithm 37:**
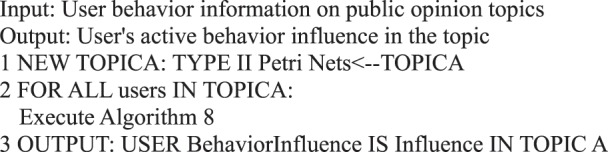
(Single-topic User Active Behavior Influence).

**Algorithm 38:**
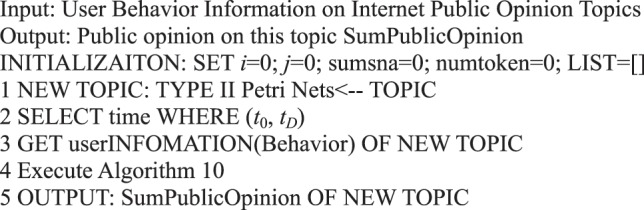
(Single Topic Public Opinion Situation).

**Algorithm 39:**
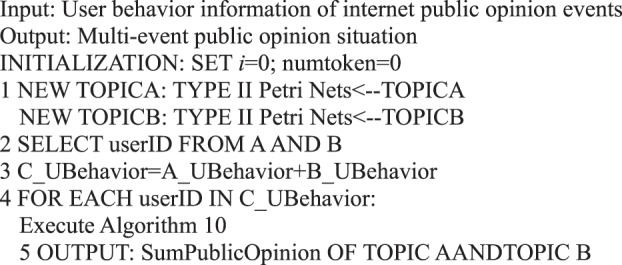
(Multi-topic Public Opinion Situation).

The proposed formal representation of Petri nets can describe the information dissemination of micro-blog flexibly and conveniently by focusing on users, overall information situation, network structure, and different information dissemination units.

## Experimental

**Figure 5:**
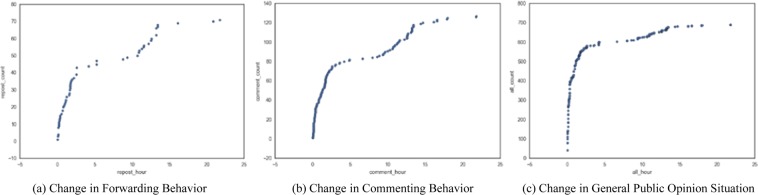
Public opinion situation of single Sina Weibo information dissemination. (**a**) Change in Forwarding Behavior. (**b**) Change in Commenting Behavior. (**c**) Change in General Public Opinion Situation.

We conduct a public opinion situation analysis as an example to illustrate the algorithm in this study. The data used in this experiment are the Weibo user data obtained through Sina’s open API interface. We use the non-relational database MongoDB to store the microblog user data, and retrieve the information based on the MongoDB storage to obtain the information needed for the experiment. The data used for public opinion situation analysis include user ID, user name, and user’s participation in a micro-blog message. For a micro-blog message, we obtain the forwarding, liking, commenting, and replying information from the publication time to 24 h after publishing the micro-blog. On the basis of the definition of the proposed public opinion situation and the formal representation of Type II Petri nets, the public opinion situation of the micro-blog information within 0–24 h is calculated. We analyze the public opinion situation of single Sina Weibo information dissemination data and obtain the change in the number of retransmitting, forwarding, and commenting behaviors and the number of replying and liking behaviors within 0–24 h after releasing Weibo information. Using Algorithm 10, we calculate the public opinion situation at every moment and obtain the change situation of the forwarding and commenting behaviors and the general public opinion situation of the micro-blog information, as exhibited in Fig. 5(a–c).

In Fig. 5, the abscissa is expressed in hour, and the ordinate represents the total number of user actions that participate in the microblog information. In Fig. 5(a), the forwarding behavior of users who participate in the transmission of the micro-blog information rapidly increases within 5 h. The total change of users’ forwarding behavior is significantly reduced 5 h after releasing micro-blog information. After being published for 24 h, the total number of forwarding actions has reached 70. In Fig. 5(b), the change in the total situation of the commenting behavior of users who participate in the micro-blog information transmission is close to the trend of participating in the forwarding behavior. This condition is due to the users who participate in the forwarding also participate in the commenting of microblog information. Moreover, 24 h after the launch, the total number of comments on the micro-blog information has exceeded 120. In Fig. 5(c), the change in public opinion is based on a user’s behavior. In accordance with the formal representation algorithm of Petri nets, Fig. 5(c) displays the instantaneous public opinion situation of this micro-blog information within 0–24 h after its release. Within 0–5 h after the release, the trend of public opinion has considerably increased and slowly changed. After24 h, the public opinion situation of the micro-blog information has reached 700.

In fact, we can draw three pictures of the change in the forwarding and commenting behaviors and the general public opinion situation of the micro-blog information in every community when we first divided the micro-blog network into communities. For a critical path in this micro-blog network, we can calculate the changes in the forwarding and commenting behaviors on the micro-blog information and the general public opinion situation on this path. Similarly, we can determine all the infected nodes of this micro-blog in a given time in chronological order. In particular, the formal representation of Type II Petri nets is convenient and flexible, thereby providing formal support for many future studies. This formal tool can aid researchers in various application fields to express many complex logic and algorithms.

In addition, many of the abovementioned models, such as independent cascade, linear threshold, and virus propagation, can describe their work based on the proposed unified formal representation. For the linear threshold model, the active and inactive states of user node *v* in the network are recorded as repositories *s*_1_ and *s*_2_, respectively. When node *v* remains unaffected by the active nodes around it to a specified threshold, user node *v* is in an inactive state, that is, repository *s*_1_. At time *q* of information propagation, when the influence of active nodes on node *v* reaches the threshold, node *v* changes from inactive state *s*_1_ to active state *s*_2_ at *q* + 1, that is, from repository *s*_1_ to repository *s*_2_. Moreover, the change from repository *s*_1_ to *s*_2_ is a user behavior process called transition *t*. The occurrence condition of transition *t* is that the influence of active nodes on node *v* reaches a specified threshold. Node *v* becomes active after the transition occurs, and node *v* in an active state can continue to exert a certain influence on other inactive nodes.

The SIR model is a typical virus propagation model. In the SIR model, users have three states in the network, namely, vulnerable state S, infectious state I, and recovery state R. These states are recorded as repositories *s*_1_, *s*_2_, and *s*_3_, correspondingly. Individuals in vulnerable state S (repository *s*_1_) change to infected state I (repository *s*_2_) with a fixed probability *β*. The change from State S to State I is called transition *t*_12_. Individuals in State I (repository *s*_1_) will recover with probability *γ* and maintain recovery state R (repository *s*_3_). The change process from State I to State R is called transition *t*_23_.

## Conclusion

To solve the problem of information dissemination in social networks, based on the structure of micro-blog network and the characteristics of users’ behavior during information dissemination and combining with the relevant theory of Petri nets, this study presents a unified formal expression method of information dissemination in a micro-blog network based on Petri nets and establishes various formal expression methods of Petri nets, such as Types I and II.

First, this study describes the information dissemination of a micro-blog network from the perspective of users’ behavior (including posting or forwarding, commenting, replying, liking, deleting, and not taking any action) and the changes between users’ behavior states. Then, the formal representation method of basic information dissemination based on Type I Petri nets is established on the basis of the change in a user’s behavior state. A unified formal representation of Type II Petri nets with time factor is established. The application of Petri net formalized representation with time factor in a micro-blog network is discussed in detail. The application includes establishing user’s social network addiction, calculating the influence of a user’s behavior, and conducting a public opinion situation analysis based on a user’s behavior. The related algorithms of the formal representation of information dissemination in a micro-blog network based on Petri nets are given, and some algorithms are analyzed through experiments.

The proposed formal representation method of information dissemination in a micro-blog network based on Petri nets is in accordance with user behavior during information dissemination, thus integrating the characteristics of Petri nets and utilizing the Petri net features, such as describing the running state of the system, representing the characteristics of the change in individual behavior state, and establishing a unified formal representation of information dissemination in a micro-blog network based on user behavior from the perspective of system operation. This unified formal method provides some support for user behavior analysis and public opinion monitoring and presents a new perspective and basis for other methods of information dissemination in social networks.

Given space limitation, this study only discusses the formal representation of Type II Petri nets. Future study will investigate many formal representation methods of Petri net micro-blogging, such as Type III. Therefore, we will provide many profound and enlightening conclusions on user behavior in social networks based on numerous achievements of Petri nets, such as delay, color, and discrete event Petri nets.
